# Lipid-Laden Alveolar Macrophages and pH Monitoring in Gastroesophageal Reflux-Related Respiratory Symptoms

**DOI:** 10.1155/2012/673637

**Published:** 2012-02-01

**Authors:** R. Kitz, H. J. Boehles, M. Rosewich, M. A. Rose

**Affiliations:** Pulmonology, Allergy and Cystic Fibrosis, Children's Hospital, Goethe University Frankfurt, Theodor Stern Kai 7, 60590 Frankfurt, Germany

## Abstract

Lipid-laden alveolar macrophages and pH monitoring have been used in the diagnosis of chronic aspiration in children with gastroesophageal reflux (GER). This study was conducted to prove a correlation between the detection of alimentary pulmonary fat phagocytosis and an increasing amount of proximal gastroesophageal reflux. It was assumed that proximal gastroesophageal reflux better correlates with aspiration than distal GER. Patients from 6 months to 16 years with unexplained recurrent wheezy bronchitis and bronchial hyperreactivity, or recurrent pneumonia with chronic cough underwent 24-hour double-channel pH monitoring and bronchoscopy with bronchoalveolar lavage (BAL). Aspiration of gastric content was determined by counting lipid laden alveolar macrophages from BAL specimens. There were no correlations between any pH-monitoring parameters and counts of lipid-laden macrophages in the whole study population, even when restricting analysis to those with abnormal reflux index expressing clinically significant GER. Quantifying lipid-laden alveolar macrophages from BAL in children with gastroesophageal-related respiratory disorders does not have an acceptable specificity to prove chronic aspiration as an underlying etiology. Therefore, research for other markers of pulmonary aspiration is needed.

## 1. Introduction

Gastroesophageal reflux is associated with a variety of respiratory symptoms from infancy and childhood [1] to adults [2], and may manifest by respiratory symptoms alone [3, 4].

In a birth cohort study, the association of heartburn and acid regurgitation to asthma symptoms increased with age [5]. Thus, looking for symptoms of GER solely in children with unexplained respiratory disorders may underdiagnose GER-related disease. Children and adults may not only present asthma symptoms, but also recurrent pneumonia, chronic cough, and signs of upper airway involvement [6, 7]. Thus, aspiration of gastric content into the airways may play a relevant role. This phenomenon is called microaspiration [8] and evidence supports this event as the most common etiology of laryngopulmonary symptoms [9].

Therefore pH monitoring of the esophagus and detection of lipids in alveolar macrophages may be useful in the diagnosis of chronic aspiration from gastroesophageal reflux in children with otherwise unexplained chronic lung disease.

Accumulation of lipids by phagocytosis of alveolar macrophages is considered to be suggestive for chronic aspiration [10, 11].

The knowledge of using lipid-laden alveolar macrophage (LLAMs) in diagnosing chronic aspiration came from animal studies: Colombo and coworkers followed rabbits with experimental aspiration of milk [12]. They found a short-time increase of LLAMs after a single instillation of milk into the trachea whereas daily repeated instillation of milk for five days lead to detection of LLAMs for the two following weeks. In a control group with saline instillation, no LLAMs could be found.

A number of further studies looked for LLAMs in BAL of patients suspicious for GER-related chronic lung disease, but studies comparing BAL results with pH monitoring of the esophagus are rare, sample sizes are small, and results contrary [13–15]. 

The hypothesis of our present study was that reflux episodes into the proximal esophagus should correlate with aspiration of gastric content, that is, the more reflux into the proximal esophagus, the more phagocytosis of reflux content in bronchoalveolar macrophages. Therefore, counting LLAM in BAL of children with chronic lung disease would correlate with pH-monitoring parameters in the esophagus.

## 2. Methods

### 2.1. Study Population

Our retrospective study surveys 448 six-month to 16-year-old children (median 4.12 years, range 0.6–16 years). The study was performed over a time period of three years from 1999–2002. Children were referred for evaluation of recurrent wheezing bronchitis and recurrent pneumonia, related to chronic cough. Symptoms had to persist for at least one year. Exclusion criteria for participation were cystic fibrosis, immunodeficiency, severe neurological disease, and relevant aeroallergic sensitizations, chronic foreign body aspiration, and pulmonary malformations. When appropriate tests were performed, bronchoscopy and pH-monitoring were performed as elective routine clinical work up in a disease-free period: patients underwent diagnostic bronchoscopy in general anesthesia. Bronchoalveolar lavage was performed in the right middle lobe or the lobe most affected by the underlying disease. Three times 1 ml/kg body weight of 0.9%, body warmed, saline solution was instilled and gently suctioned. Lavage fluid was centrifuged with 1000 rpm for 10 min with a cytospin 2000 (Shandon, Southern Products, UK). Oil-Red-O-stained slides were examined microscopically for lipid phagocytosis into alveolar macrophages, consequently called “lipid-Laden alveolar macrophages” (LLAMs). 

**Figure 1:**
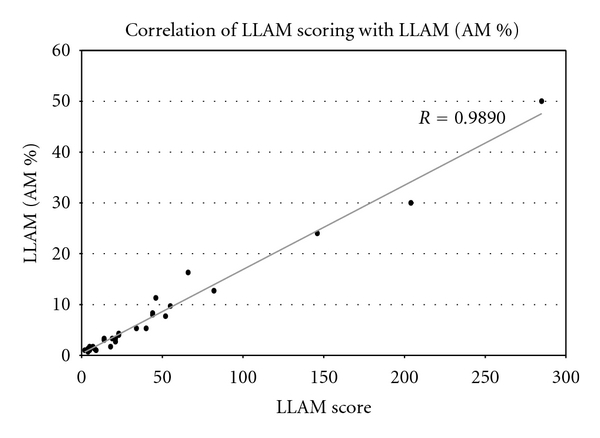
Pearson's correlation of LLAM scoring with LLAMs (&AMs). High correlation of the percentage of fat containing AMs (LLAMs/%AMs) (from a total of 300 screened AMs) and their fat content described by the LLAMs score.

In a pilot study, we compared counting LLAMs and expressing them as a ratio of the total amount of alveolar macrophages (AMs) and compared this ratio to the scoring LLAM-index method of Colombo [16]. Briefly with this method a total of 300 alveolar macrophages were screened for cytoplasmatic lipid granules, and AMs were graded to their content of lipid stained: 0 = cytoplasm not opacified, 1 = up to 1/4 opacified, 2 = up to 1/2 opacified, 3 = up to 3/4 opacified, and 4 = totally opacified cytoplasm. Thus, LLAM scores may range from 0 to 1200. Counting LLAMs may result in ratios of 0–100% of 300 AMs. Researchers who scored the LLAMs were blinded to the pH-monitoring results. Our pilot study had shown a good correlation between the ratio of LLAM/AM and the Colombo method of qualitative scoring LLAM (Pearson's *r* = 0.9890). For details see Figure 1. Thus, we could use this ratio of LLAM/AM count for further evaluation in the study.

### 2.2. Monitoring of pH

In a timely manner to the bronchoscopy, patients underwent double-channel 24 hr esophageal pH monitoring according to the ESPGHAN standardized protocol [17]. None of the subjects had received antireflux treatment before. A monocrystalline antimony double-channel pH probe (Medtronic Synectics Medical, Sweden) was calibrated in buffers of pH 7.01 and 1.07 and then placed transnasally into the esophagus. The distance of the two pH sensors on the probe was adjusted according to the patient's body height, using our previously published formula [18]. The upper probe position of the sensors was always chosen to be set at the line between the clavicles. The lower probe position was set in the distal esophagus.

Therefore, we used three different distances between the sensors: 5 cm in patients with a body height of ≤80 cm, 10 cm in patients with a body height of ≤120 cm, and 15 cm in patients with a body height of >120 cm. This facilitates interindividual comparability for esophageal pH-monitoring. After adjusting the probe for an optimal position within the esophagus using a chest X-ray, probes were connected to a portable digital recorder (Digitrapper MK III, Synectics Medical AB, Sweden). Patients and their guardians were then asked to keep a diary for the next 24 hours. This diary included data on the time and kind of consumed meals, beverages, and drugs as well as posture. After 24 hours, data were analyzed by the software “Esophogram” (Synectics Medical AB, Sweden). Reflux episodes were defined as a decrease of esophageal ph below 4 for longer than five seconds, followed by an increase of pH for a minimum of 4.5, thus avoiding oscillating phenomena. This enabled us to detect the number of reflux episodes at each sensor in the past 24 hours, the longest reflux episode, and the number of long-lasting reflux episodes (>5 minutes). The summarized time of all reflux episodes divided by the total recording time (in general over a period of 24 hours) is also known as the “reflux index.”

### 2.3. Statistical Analysis

Mean values were given as medians ± ranges. Linear regression analysis (Pearson's correlation coefficient) was used to assess the correlation between pH-monitoring parameters and percentage of lipid-laden alveolar macrophages (LLAMs). A probability value of *P* < 0.05 indicated statistical significance between groups. Testing was performed using SPSS 11.0 for windows (SPSS Inc., Chicago, Illinois USA).

## 3. Results

**Figure 2:**
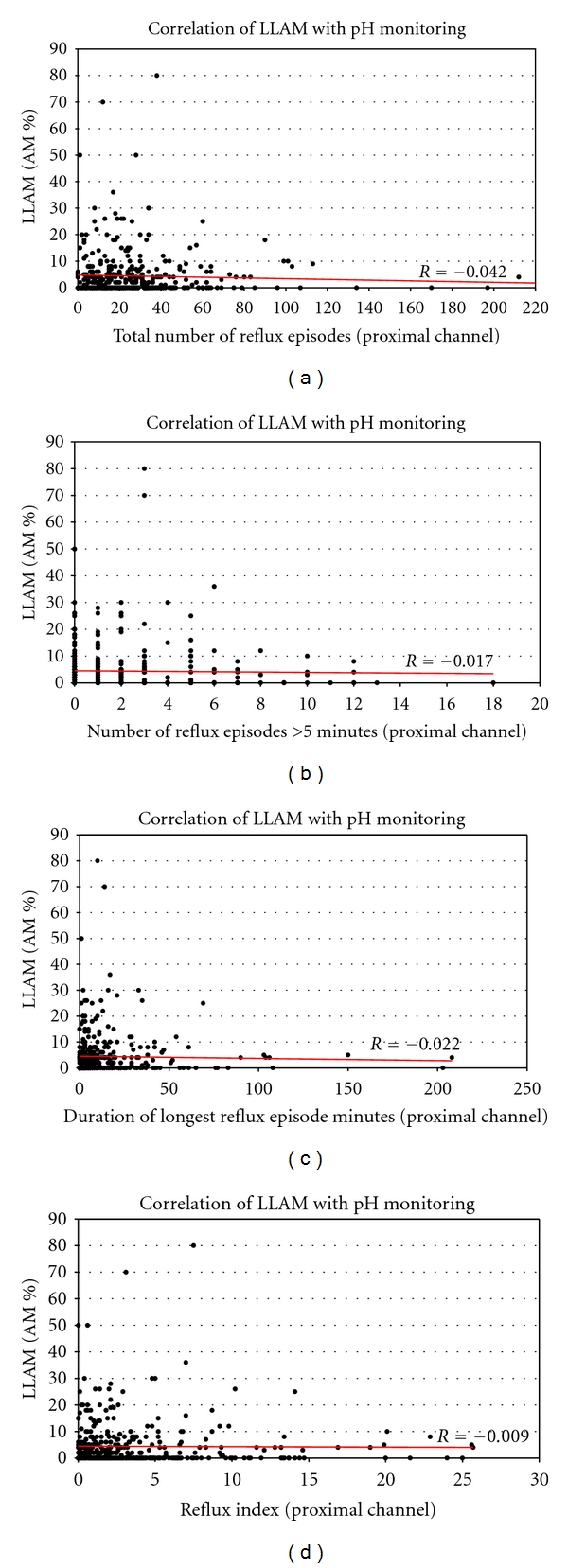
Scatter plots of parameters from the proximal channel of a double-channel 24 hr pH probe in the esophagus versus LLAM count (in % of 300 AMs; *n* = 448). (a) Total number of reflux episodes: no correlation with the percentage of fat containing AMs (LLAMs/%AMs) in the regression analysis (*r* = −0.042). (b) Number of reflux episodes, lasting longer than five minutes: no correlation with the percentage of fat containing AM (LLAMs/%AMs) in the regression analysis (*r* = −0.017). (c) Duration (minutes) of the longest reflux episode: no correlation with the percentage of fat containing AMs (LLAMs/%AMs) in the regression analysis (*r* = −0.022). (d) Reflux Index (percentage of time pH below 4): no correlation with the percentage of fat containing AMs (LLAMs/%AMs) in the regression analysis (*r* = −0.009).

Percentages of lipid-laden alveolar macrophages in BAL (LLAM/AM) were correlated to esophageal pH-monitoring parameters. No correlations could be revealed between the ratio of LLAM/AM and results of pH-monitoring parameters at the proximal channel of the pH-probe linear regression analysis (Pearson's correlation coefficient) of the number of reflux episodes (*r* = −0.042), the number of reflux episodes lasting longer than 5 minutes (*r* = −0.017), the longest reflux episode (*r* = −0.022) and at last the reflux index (*r* = −0.009) did not show a significant correlation to LLAMs as the proposed marker of aspiration (see Figures 2(a), 2(b), 2(c), and 2(d)). This was also true when correlating pH values from the distal channel to the ratio of LLAM/AM (data not shown).

Further investigation was restricted to patients with presumed GER, indicated by positive pH-monitoring results. Their reflux index needed to be ≥5% at the distal channel (*n* = 164). In these patients, mean ratio of LLAM/AM was 5.04% whereas mean ratio of LLAM/AM in the other patients without GER (RI < 5%) at the distal channel was 4.3%. The results missed statistical significance (see Table 1).

Again, there were no correlations between results of pH-monitoring parameters (e.g., number of reflux episodes, reflux episodes lasting longer than five minutes, the longest reflux episode, and reflux index) and the ratio of LLAM/AM counts in both subgroups (data not shown). 

Despite more reflux activity in the pH-monitoring results in children younger than four years of age, we did not detect a correlation of age to parameters of pH monitoring and ratio of LLAM/AM than in patients four to 16 years of age (data not shown).

## 4. Discussion

Our study was done to examine the predictive value of pathological findings from pH monitoring and the detection of LLAMs as to chronic aspiration in gastroesophageal reflux. We hypothesized that an increasing amount of refluxes detected at the proximal tube of the pH monitoring was indicative for pulmonary aspiration detected by LLAMs. The weak correlation between values from pH monitoring and the LLAM scores proved the null hypotheses. Nonetheless, the results from our study have to be interpreted with the restriction that lipids incorporated by alveolar macrophages are not exclusively observed in pulmonary aspiration. Fat in macrophages can also be of endogenous origin as suggested by studies on acute pneumonia [19] and in fat infusions [20]. Therefore, in gastroesophageal reflux, lipid-laden alveolar macrophages do not definitely discriminate aspirators from nonaspirators. Another possible confounder is the half-life of lipids in alveolar macrophages, which is only known from animal models [12]. In patients with pathological pH-measurements, aspiration might have taken place before pH-probe measurements were performed, resulting in false-negative results.

We nevertheless believe that LLAMs have their role in the differential diagnosis of pulmonary aspiration. Even the absence of a correlation does not ban LLAMs as a diagnostic tool. Vice versa, pH monitoring might not provide relevant results as to the underlying pathology of GER. As we know from impedance measurements of the esophageal motility, also alkaline refluxes or an insufficiency of the upper esophageal sphincter might be responsible for the aspiration. Here, proximal refluxes will only contribute to the risk of aspiration, and with a sufficient upper sphincter function, reflux episodes will not result in aspiration.

Over all, the specificity and sensitivity for the detection of lipids in alveolar macrophages appears not high enough for its sole use as a clinical decision tool. Additional markers of aspiration (e.g., pepsin, radioactive tracers such as Technetium-99m, or food additives might better provide evidence for recurrent aspiration and its role in chronic lung disease [9, 21, 22]).

## 5. Conclusion

This study has shown that counting or scoring of lipid-laden alveolar macrophages from bronchoalveolar lavage is not a reliable tool to solely test for chronic aspiration diagnosed by esophageal pH monitoring in children with predominantly asymptomatic gastroesophageal reflux. Other markers of aspiration are needed for clinical practice to prove the causal relationship between gastroesophageal reflux and chronic aspiration. These markers should be of exogenous origin to enhance specificity of the test by avoiding endogenous interferences. Other tools to better detect GER (apart from esophageal pH monitoring) should be introduced to cover also non acid reflux (e.g., multilocal impedance measurements of the esophagus).

## Figures and Tables

**Table 1:** Regression analysis from pH-monitoring parameters correlated with LLAMs (% AMs) from BAL with patients stratified for RI > 5% or RI < 5% at distal channel recordings. No correlations could be found in these subgroups. *P* values indicate no statistical differences for these correlations in patients with RI < 5% at distal channel recordings (n.s. = not significant).

	No. of reflux episodes	No. of reflux episodes >5 min.	Longest reflux episode	Reflux index
Ratio (%) LLAM/AM	*r* = 0.228	*r* = −0.0264	*r* = −0.11	*r* = −0.0387
	n.s.	n.s.	n.s.	n.s.

## Abbreviations

AM: Alveolar macrophage
LLAM: Lipid-laden alveolar macrophage
GER(D): Gastroesophageal reflux (disease)
BAL: Bronchoalveolar lavage
RI: Reflux index.
